# Data-driven combination of METAR observations and CAMS reanalysis aerosols to enhance satellite retrieval of surface solar irradiance

**DOI:** 10.1038/s41598-026-39971-w

**Published:** 2026-02-16

**Authors:** Arindam Roy, Detlev Heinemann, Marion Schroedter-Homscheidt, Jorge Enrique Lezaca Galeano

**Affiliations:** 1 German Aerospace Center (DLR), Institute of Networked Energy Systems, Energy System Analysis, Oldenburg, Germany; 2 University of Oldenburg, Institute of Physics, Oldenburg, Germany

**Keywords:** Satellite-estimated solar irradiance, Aerosol, Classical and quantum learning, CAMS, McClear, METAR, Climate sciences, Environmental sciences

## Abstract

Accurate solar irradiance forecasts are vital for photovoltaic (PV) power prediction, especially in tropical and subtropical regions affected by dust, wildfire smoke, and pollution. Yet, aerosol detection from satellites is often obstructed by clouds, AErosol RObotic NETwork (AERONET) stations are sparsely distributed, and climatological datasets cannot capture intra-day variability. Global products such as the Copernicus Atmosphere Monitoring Service (CAMS) provide broad coverage but miss local events due to coarse resolution and uncertainties in the underlying emission database. In this study, atmospheric parameters from automated METeorological aerodrome report (METAR) observations and CAMS aerosol products are used as inputs to data-driven models trained on normalized pseudo global horizontal clear sky irradiance (${GHI}_{CS}^{*}$) targets from one site. Models tested include gradient boosting methods, Random Forests, neural networks, and a quantum variational circuit. Results have been obtained using only openly available data from seven test sites with significant aerosol loads, for the period spanning 2015–2024. The predicted global horizontal clear sky irradiance (${GHI}_{CS}$) is then used in the Heliosat-3 method, which uses satellite-derived cloud index (CI) to estimate the all-sky global horizontal irradiance (GHI), for benchmarking against the all-sky GHI output of Heliosat-3 coupled with ${GHI}_{CS}$ from the physics-based McClear model. Categorical boosting (CatBoost) shows the highest positive root mean squared error (RMSE) skill score (SS) of 4.2% over the entire test dataset, compared to the reference McClear. A consistent positive RMSE SS from 1–5% is observed for the 6–8 km visibility range for all models. During dust and sand events, the Light Gradient-Boosting Machine (LightGBM) shows a 21% positive RMSE SS. These findings demonstrate the value of ${GHI}_{CS}^{*}$ based machine learning approach for improving solar irradiance estimates in aerosol-rich environments.

## Introduction

The integration of solar energy into the electricity grid presents unique challenges due to the fluctuating nature of solar irradiance, which can significantly affect power generation and grid stability. Accurate day-ahead and intra-day forecasts of all-sky global horizontal irradiance (GHI) are therefore essential: they support power system scheduling, reduce balancing costs, and help photovoltaic (PV) operators avoid penalties arising from forecast–production mismatches^1–3^. While day-ahead forecasts typically rely on numerical weather prediction (NWP), intra-day corrections are often derived from geostationary satellite imagery, which better provides more accurate cloud information due to the higher resolution^4,5^. Clouds remain the dominant source of irradiance variability^6^, but extreme aerosol events—such as dust storms, biomass burning, or urban smog—can also cause GHI reductions comparable to cloud cover^7–9^. These effects are particularly significant in tropical and subtropical regions, including the Indian subcontinent, eastern China, and Indochina, where some of the highest PV deployment rates coincide with frequent aerosol episodes^10–12^.

Estimating global horizontal clear-sky irradiance (${GHI}_{CS}$) is a critical step for satellite-based all-sky GHI retrieval. Conventional approaches rely on aerosol optical depth (AOD) inputs for radiative transfer or empirical models^13,14^. Information on atmospheric aerosol concentration can be obtained at different spatio-temporal resolutions from satellite observations, numerical modelling, ground measurements or climatological datasets^15–18^. However, aerosol information is imperfect across all available sources. Satellite retrievals are limited by cloud contamination, choice of aerosol model and assumptions about aerosol properties^19^. Ground-based networks such as AERONET provide high-quality AOD measurements, but coverage is sparse and point-based observations are often unrepresentative^20,21^. Climatological datasets cannot capture rapid intra-day aerosol fluctuations^18^. A widely used tool for ${GHI}_{CS}$ estimation is the McClear model^22^, which computes ${GHI}_{CS}$ using AOD and other inputs from the Copernicus Atmosphere Monitoring Service (CAMS). McClear has been shown to perform well under many conditions globally^23^. However, it inherits the limitations of the CAMS aerosol data. CAMS provides global, hourly, 40 km–resolution fields and offers valuable large-scale coverage, but its spatial and temporal resolution makes it less suited to representing local or short-lived aerosol events. Regional assessments have reported systematic biases, such as underestimation of AOD in high-load conditions in Australia^24^, misrepresentation of fine-mode aerosols over the Indo-Gangetic Basin^25^, and inconsistencies in regions strongly influenced by biomass burning, desert dust, or mixed aerosol sources including Brazil and the Eastern Mediterranean^26–28^. Therefore, the following uncertainties can be identified with the different sources of aerosol information: (i) limitations in retrieval and numerical modelling algorithms, (ii) naïve aerosol constancy assumptions in climatology, and (iii) limited representativeness of sparsely available ground measurements.

Surface horizontal visibility has long been recognized as a proxy for aerosol extinction^29–35^, with early work such as the Elterman model^34^ establishing a link between visibility and vertical aerosol profiles. Modern retrievals have refined these methods with empirical corrections, optimization techniques, and calibration against satellite AOD products^36–40^. Although vertical visibility is more closely related to AOD, it is only reported when obscuring phenomena or ceiling conditions require a vertical measure, as recommended by WMO^41^. In contrast, horizontal visibility is routinely reported in METeorological Aerodrome Reports (METAR) at airports worldwide^42^, yielding a dense, near-real-time dataset that far surpasses the spatial coverage of dedicated aerosol networks^43–45^. Therefore, due to the sparse availability of vertical visibility measurements, it is not considered in this study and the term “visibility” henceforth will refer to horizontal visibility specifically. Visibility is influenced not only by aerosols but also by humidity, fog, precipitation, and wind^46–48^. As a result, its correlation with ground-based AOD is modest except under dust-dominated conditions^49,50^, and the interaction between AOD and relative humidity (RH) further complicates the relationship^51,52^. On its own, visibility is therefore insufficient as a direct substitute for AOD, but it holds promise when integrated with complementary datasets.

Machine learning (ML) offers a flexible framework for combining heterogeneous inputs and extracting non-linear relationships that elude traditional parameterizations^53,54^. Previous studies have applied tree-based models such as decision trees and random forests to estimate visibility from monthly or daily aerosol information and vice-versa^43,54^, but they fail to resolve rapid aerosol changes, leading to biased irradiance forecasts^55,56^. More advanced ML methods, including gradient boosting frameworks (e.g., XGBoost, LightGBM, CatBoost) and Neural Networks, offer improved performance, scalability, and robustness across diverse datasets^57–61^. Quantum variational circuits (QVCs) have also been proposed for ML applications^62,63^, though their application to solar energy meteorology remains largely exploratory. Despite these advances, few studies have systematically explored the integration of real-time visibility (METAR) with reanalysis products (CAMS) to improve clear-sky irradiance estimation.

This study addresses that gap with a data-driven framework for estimating ${GHI}_{CS}$. Specifically, It:

1. Presents a data-driven approach using machine learning (ML) models for estimating global horizontal clear sky irradiance (${GHI}_{CS}$) by combining METAR and CAMS aerosol datasets.
2. Presents an approach for obtaining normalized pseudo global horizontal clear sky irradiance (${GHI}_{CS}^{*}$) targets using ground measured GHI, satellite estimated cloud index (CI) and the top of atmosphere (${I}_{TOA}$) irradiance, in order to compensate for the lack of direct measurements of ${GHI}_{CS}$ in all-weather situations.
3. Benchmarks the accuracy of satellite-estimated all-sky GHI derived using the ${GHI}_{CS}$ output from the ML models utilizing METAR and CAMS data against the satellite-estimated all-sky GHI derived using the ${GHI}_{CS}$ McClear model, at seven unseen sites.
4. Validates the improvement in estimated all-sky GHI across a range of visibility situations and aerosol-related METAR weather codes.
5. Validates the improvement in estimated all-sky GHI across a range of RH conditions.

## Data and method

### Ground measured GHI

Ground observations of GHI are obtained from eight stations located in regions strongly influenced by diverse aerosol conditions (Table 1). Data for Cairo, Gurgaon, Da Nang, Chiba, Adrar, Ghardaia and Pretoria are obtained from the CAMS Evaluation and Quality Control database hosted at Mines Paris^64^, while Xianghe measurements are retrieved via the BSRN FTP server^65^. The Cairo, Adrar, Ghardaia and Xianghe stations are equipped with Kipp & Zonen CMP21 secondary standard class A pyranometers, Gurgaon uses an Eppley PSP pyranometer, Da Nang is equipped with Huskeflux SR20 secondary standard class A pyranometer, Pretoria has a CMP11 secondary standard pyranometer and the Chiba SKYNET station employs a POM-01 sky radiometer. Table 1 provides a summary of the GHI measurement stations used in this study. All datasets are quality controlled using the open-source libinsitu software package developed under the International Energy Agency – Photovoltaic Power Systems Programme (IEA-PVPS) Task 16^64,66^. This includes removal of values flagged as invalid by the physical possible limit (PPL) and extremely rare limit tests. Following quality control, the GHI datasets are averaged from 1-min to 30-min resolution before being used in this study.

**Table 1:** Stations providing GHI ground observations.

Site	Network	Location	Source of data	Time period	Native temporal resolution (min)	Distance to next airport/METAR observation (km)
Cairo	enerMENA	30.04 ˚N, 31.01˚E	http://tds.webservice-energy.org/	2015–2019	1	39
Gurgaon	BSRN	28.42 ˚N, 77.16 ˚E	http://tds.webservice-energy.org/	2018–2019	1	12
Da Nang	ESMAP	16.01 ˚N, 108.19 ˚E	http://tds.webservice-energy.org/	2017–2019	1	8
Xianghe	BSRN	39.75 ˚N, 116.96 ˚E	ftp://ftp.bsrn.awi.de/	2010–2015	1	71
Chiba	SKYNET	35.63 ˚N, 140.10 ˚E	http://tds.webservice-energy.org/	2015–2017	1	20
Adrar	enerMENA	27.88 ˚N, 0.27 ˚W	http://tds.webservice-energy.org/	2015–2016	1	9
Ghardaia	enerMENA	32.39 ˚N, 3.78 ˚E	http://tds.webservice-energy.org/	2015–2018	1	2
Pretoria	SAURAN	25.75 ˚S, 28.23 ˚E	http://tds.webservice-energy.org/	2015–2024	1	9

These stations are selected because they are located in regions characterized by frequent and diverse aerosol loading:

#### Cairo

Strongly affected by a mix of urban emissions, biomass burning, and desert dust^67^. Dust storms, especially in spring, contribute to high AOD and influence cloud properties^68^. A unique “urban haze” composed of submicron ammonium chloride (from biomass burning) and super micron dust has been reported^69^.

#### Gurgaon (near Delhi)

High aerosol concentrations result from industrial-vehicular emissions, biomass burning and dust storms, with significant seasonal variations. Biomass burning dominates in the post-monsoon and winter periods^70,71^, industrial emissions persist year-round with peaks after monsoon^72^ and dust storms are common during pre-monsoon and monsoon^73^.

#### Xianghe (near Beijing)

Summer exhibits the highest AOD and fine-mode fraction due to urban haze^74^, winter has moderate AOD with increased coarse-mode aerosols from heating activities^75^, and spring is influenced by desert dust^74^.

#### Chiba (near Tokyo)

Organic aerosols dominate composition (40 – 60%) across seasons, with daytime peaks^76^. Diesel exhaust is a major source of fine particulate matter^77^.

#### Da Nang

Rice straw burning during late summer-autumn harvests elevates PM_2.5_ and NO_2_^78^. Such practices are most prevalent during the harvest season from late summer to early autumn. Black carbon from quarrying and vehicular pollution peaks in the dry season (June – July)^79^.

#### Adrar

The Adrar plateau is a significant dust source region^80^. Existing studies have found elevated atmospheric turbidity in summer period due to a low cohesion of sand/dust particles caused by higher ambient temperature and lower relative humidity, accompanied with stronger winds that can transport sand and dust particles^81^.

#### Ghardaia

Hot weather and Sirocco winds result in increased dust/ sand aerosol during summer^82^. Prone to urban aerosols due to the prevalence of mining-related crusher plants in the area^83^.

#### Pretoria

Elevated atmospheric aerosol concentration due to anthropogenic sources of air pollution, particularly due to bio-fuel burning in winter^84^. Atmospheric brown clouds with large amounts of absorbing aerosols, including black carbon, are frequently observed^85^.

### Cloud observations from satellites

Surface Solar Radiation Data Set – Heliosat (SARAH-3)^86^, available at 30-min temporal resolution on a 0.05˚ × 0.05˚ regular grid, is generated by applying the MAGICSOL algorithm on the images from Meteosat Second Generation (MSG2), located at 0 ˚E. MAGICSOL derives the effective cloud albedo (CAL) using the original Heliosat method^87^.

The Heliosat method is a widely-used approach for estimating surface solar irradiance from satellite imagery, based on the relationship between cloud optical properties and solar radiation attenuation. The method exploits the principle that satellite-observed reflectance is inversely related to ground-level irradiance: bright (cloudy) pixels correspond to lower surface irradiance, while dark (clear-sky) pixels indicate higher irradiance. Heliosat-1^88^, -2^89^, and -3^90^ employ a normalization technique (shown in Eq. 1) to convert satellite-measured reflectance into CI, followed by the empirical relation in Eq. 2 to convert CI into clear-sky index ${k}_{c}$ (the ratio of GHI to $GH{I}_{CS}$). GHI is subsequently derived by multiplying ${k}_{c}$ with $GH{I}_{CS}$ from a clear sky model. These empirical versions differ primarily in the calibration of reference surface albedo ${\rho }_{g}$ and cloud albedo ${\rho }_{c}$. However, they all share the same fundamental concept of estimating atmospheric transmittance through satellite-observed reflectance.

**Table 2:** Satellite-estimated products used in this study.

Site name	Satellite product name	Source
Cairo	Cloud albedo	Online repository of the Satellite Application Facility (CM-SAF) on Climate Monitoring, SARAH-3 dataset
Adrar	Cloud albedo	Online repository of the Satellite Application Facility (CM-SAF) on Climate Monitoring, SARAH-3 dataset
Ghardaia	Cloud albedo	Online repository of the Satellite Application Facility (CM-SAF) on Climate Monitoring, SARAH-3 dataset
Pretoria	Cloud albedo	Online repository of the Satellite Application Facility (CM-SAF) on Climate Monitoring, SARAH-3 dataset
Gurgaon	Cloud opacity	Solcast web platform and API
Da Nang	Cloud opacity	Solcast web platform and API
Xianghe	Cloud opacity	Solcast web platform and API
Chiba	Cloud opacity	Solcast web platform and API

1 $$\begin{array}{c}CI=\frac{\left(\rho -{\rho }_{g}\right)}{\left({\rho }_{c}-{\rho }_{g}\right)}\end{array}$$

In the SARAH-3 dataset, CAL is the variable corresponding to CI. For this study, CAL values for the Cairo station (30.04 ˚N, 31.01 ˚E) are extracted via spatial interpolation for the time period 2015–2019 (Table 1), which corresponds to the openly available reference data from the stations under IEA-PVPS Task 16. Similarly, CAL values for Adrar, Ghardaia and Pretoria (Table 2) are also extracted via spatial interpolation for the respective time periods mentioned in (Table 1). CAL is converted to clear sky index (${k}_{c}$) following the procedure in^91^, summarized in Eq. (2).

2 $$\begin{array}{c}{k}_{c}=\left\{\begin{array}{c}1.2, {\mathrm{f}}{\mathrm{o}}{\mathrm{r}} CI\le -0.2\\ 1-CI, {\mathrm{f}}{\mathrm{o}}{\mathrm{r}}-0.2\le CI\le 0.8\\ 1.661-1.7814CI+0.7250{CI}^{2}, {\mathrm{f}}{\mathrm{o}}{\mathrm{r}} 0.8\le CI\le 1.05\\ 0.09, {\mathrm{f}}{\mathrm{o}}{\mathrm{r}} 1.05<CI\end{array}\right\}\end{array}$$

where,

${k}_{c}:$ clear sky index

$$CI:$$ cloud index

Complementary datasets of cloud opacity at 30-min resolution for Xianghe, Chiba, Gurgaon and Da Nang (Table 2) are obtained from the Solcast platform^92^. Solcast does not release the full details of its proprietary methodology; however, published studies indicate that its approach is based on semi-empirical retrievals of cloud properties from geostationary satellite imagery^93^. In line with prior literature^94^, cloud opacity is considered equivalent to CI (or CAL), and is therefore converted to ${k}_{c}$ using Eq. ($2$).

### Aerosols and other atmospheric parameters

#### McClear clear sky irradiance and CAMS aerosol

${GHI}_{CS}$ For the sites used in this study are obtained from the McClear service of CAMS^22^. The atmospheric composition input into the McClear model comes from the CAMS EAC4 global reanalysis prior to 2023–07^95^, which has a spatial resolution of 0.75° × 0.75° and a temporal resolution of 3 h. The McClear model uses inputs from the CAMS global atmospheric composition forecast from 2023–07 onwards, which has a spatial resolution of 0.35° × 0.35° and a temporal resolution of 3 h^96^. In addition, McClear internally calculates solar geometry parameters and top of atmosphere irradiance (${I}_{TOA}$). For this study, McClear ${GHI}_{CS}$ and the same atmospheric composition data used by McClear (CAMS EAC4 for the period before 2023–07 and CAMS global atmospheric composition forecasts for the period after 2023–07) are retrieved for each site with the Climate Data Store Applications Program Interface (cdsapi). Outputs are requested at 30-min temporal resolution, consistent with the temporal resolution of the METAR data. As CAMS reanalysis is available at 3 hourly resolution, the 30-min values are obtained by assuming constant atmospheric conditions within each 3 h window. The full list of parameters used in this study is summarized in (Table 3).

**Table 3:** Summary of the CAMS global reanalysis parameters used in this study.

Parameter	Description
${I}_{TOA}$	Irradiation on a horizontal plane at the top of atmosphere
sza	Solar zenith angle in degrees
tco3	Total column content of ozone in Dobson unit
tcwv	Total column content of water vapour in kg/m^2^
AOD	Total aerosol optical depth at 550 nm

#### METAR

METAR recorded atmospheric parameters observed once every 30 min are obtained for the closest airport to the eight sites. The International Civil Aviation Organization (ICAO) mandates automated visibility measurements from transmissometers or forward scatter meters for all airports which have runways where Category (CAT) II and CAT III Instrumented Landing Systems (ILS) are used^97^. For runways using ILS CAT I, automated visibility measurements are also recommended. The datasets shown in Table 4 are downloaded from the Iowa Environmental Mesonet repository^98^ maintained by the Iowa State University of Science and Technology, which has a long-term archive of airport Automated Surface/ Weather Observation Stations (ASOS/AWOS) for weather parameters. The temperature, wind speed and visibility measurements are converted to SI units, i.e., ˚C, m/s and km. Visibility measurements at airports commonly use transmissometers and forwards scatter sensors for METAR reports^99^. Quality checks involve comparing sensor data with human observations and reference instruments.

**Table 4:** Atmospheric parameters from METAR data.

Parameter	Description
relh	RH in %
vsby	Visibility in miles
wxcodes	Significant weather observations

Furthermore, METAR provides observations of the significant weather. Namely, the classes Haze (HZ), Smoke (FU), Widespread Dust (DU), Sand (SA), Sandstorm (SS), Duststorm (DS) and Dust/ Sand whirls (PO) are related to aerosols and are used for diagnostic classification of the results.

## Machine learning setup

In this study, a group of ML models (described in Sect.  3.4) are used to directly estimate a normalized pseudo global horizontal clear sky irradiance (explained in Sect.  3.3) with chosen CAMS Reanalysis and METAR parameters (described in Sect.  3.2). This essentially combines the (i) Visibility to AOD, and (ii) AOD to Clear sky irradiance conversion steps into one, and avoids the need for a separate Visibility to AOD conversion methodology.

### Training-validation-test data split

Cairo is a site with a large number of data points and is characterized both by dust and anthropogenic pollution conditions. Therefore, it is chosen for the development of the ML models.

Two-third of the available datapoints from Cairo, as shown in (Table 5) are used for training the models and the remaining one-third for validation and hyperparameter tuning. The training-validation split is not done randomly but in a chronological manner, to ensure that different datapoints from the same days do not appear in the training and validation datasets. Otherwise, due to similarity in the atmospheric situation over a day, the model may produce memorized results instead of learning. The data from the remaining seven sites are used to test the performance of the model on previously unseen sites. This is done to check whether the trained models are able to overcome site-dependency.

**Table 5:** Availability of quality controlled datapoints for the analysis.

Site	Quality checked datapoints	
	Training and validation	Testing
Cairo	20,914	-
Gurgaon	-	6,222
Da Nang	-	12,800
Xianghe	-	16,268
Chiba	-	14,682
Adrar	-	13,640
Ghardaia	-	20,430
Pretoria	-	65,560

### Predictor preparation

In order to reduce the computational load, the CAMS AOD values of the different species at 550 nm are not entered simultaneously as inputs into the models. Instead, the (i) total AOD at 550 nm is used in this analysis. Further input parameters into the ML models are selected as follows: (ii) Visibility measurements from airport, which provide local information on the atmospheric aerosol loading at the surface. (iii) RH, as it is correlated to the presence of fog and mist, which are known to occur with smog. (iv) Solar zenith angle (SZA), as the cosine of SZA is inversely proportional to the air mass that ${I}_{TOA}$ travels through and undergoes dissipation before reaching the surface. (v) Solar azimuth angle, as it is correlated to the diurnal movement of the Sun. (vi) Total column water vapour (TCWV), as it is found to be a significant contributor to the reduction of GHI and the dissipative effect increases with the increase in SZA^100^.

### Target preparation

The cloud-free component of irradiance in all-sky situations cannot be measured directly. GHI measurements taken during cloudless periods are equivalent to ${GHI}_{CS}$. Various approaches for filtering clear sky situations are found in the literature, and the majority of them uses a clear sky model or requires all three components of solar irradiance or use some statistical approaches^101–103^. While the filtering step greatly reduces the number of datapoints available for training and validation, it also risks removing events where irradiance decreases because of aerosols rather than clouds. This occurs because aerosol-driven irradiance drops can be misclassified as cloudy conditions, particularly during dust storms or smog episodes where aerosols and clouds often co-occur. Furthermore, as ${GHI}_{CS}$ is finally used for deriving satellite-estimated GHI from CI in both clear and cloudy situations, it is necessary to evaluate its performance also in both situations. Due to these reasons, no explicit separation of clear sky and cloudy sky datapoints are performed in this study. Instead, a normalized pseudo global horizontal clear sky irradiance (${GHI}_{CS}^{*}$) is derived, starting from the expression of ${GHI}_{CS}$ at ground level shown in Eq. 3.

3 $$\begin{array}{c}GH{I}_{CS}=\frac{GHI}{{k}_{c}}\approx \frac{GH{I}_{ground}}{\left(1-n\right)}\end{array}$$

where,

$GH{I}_{ground}:$ ground measured GHI

${k}_{c}:$ clear sky index

${GHI}_{CS}:$ clear sky GHI

$n$: satellite estimated cloud index or cloud opacity

$GH{I}_{ground}$ is obtained from surface measurements and CI from satellite images. Of course, this equation will not hold true for situations where the cloudiness seen by the pyranometer at the surface level does not match the cloudiness seen from satellite. This could be due to the effects of parallax and cloud shadow displacement, in which case systematic biases depending on sun position (time of day), cloud height and site location with respect to the satellite, are expected^104^. This could also be due to the limitations in cloud resolving capability because of the relatively coarse spatial resolution of satellite pixels, in which case random outliers are expected^105^. However, it is expected that the statistics-based machine learning methods will be able to handle these outlier situations. Furthermore, the above expression is normalized by ${I}_{TOA}$ in order to restrict the ${GHI}_{CS}^{*}$ values within the range $\left[\mathrm{0,1}\right]$, as shown in Eq. 4, which is more efficient for training ML models. Overshooting of GHI values beyond ${I}_{TOA}$ due to cloud enhancement are neglected in this approach, which is justified by the 30 min averages of GHI analyzed.

4 $$\begin{array}{c}Target=\frac{GH{I}_{clear}}{{I}_{TOA}}=\frac{GH{I}_{ground}}{\left(\left(1-n\right)\times {I}_{TOA}\right)}\end{array}$$

### Machine learning models

Popular models for multi-variate regression are used in this analysis, including gradient boosting methods – (i) XGBoost, (ii) LightGBM, (iii) CatBoost, tree-based methods – (iv) Extra Trees, (v) Random Forest, and (vi) Neural Network. Furthermore, a more recent approach of using QVC for machine learning has also been explored. The following subsections provide a brief description of each model.

#### EXtreme gradient boosting (XGBoost)

XGBoost leverages the principles of boosting ensemble techniques to enhance prediction accuracy. It operates on the premise of sequentially adding weak learners (typically decision trees) to improve the performance of the overall model. XGBoost employs a unique regularization approach and handles missing values internally while optimizing computation speed and model robustness through parallel processing. An efficient and scalable Python implementation of XGBoost published by the original authors has been used in this analysis^106^.

#### Light gradient-boosting machine (LightGBM)

LightGBM, developed by Microsoft, improves upon traditional gradient boosting frameworks by integrating Gradient-based One-Side Sampling (GOSS) and Exclusive Feature Bundling (EFB) techniques. These innovations allow LightGBM to handle vast datasets effectively while reducing memory usage and computation time. Similar to XGBoost, LightGBM uses a decision-tree-based learning algorithm but optimizes the training process by exclusively focusing on the gradients of the chosen data subset. The latest version of the official LightGBM python implementation from Microsoft is used in this analysis^107^.

#### Categorical boosting (CatBoost)

CaBoost is a gradient boosting algorithm that uses ordered boosting to reduce prediction shift and target leakage^108^. In several studies, CatBoost achieved competitive or enhanced accuracy on tasks with imbalanced or categorical data, although its training speed was generally slower than that of LightGBM and XGBoost^109^.

#### Random forest

Random Forests combine many decision trees to improve predictions^110^. Random Forests build trees by drawing bootstrap samples and choosing splits that optimize measures such as impurity or variance reduction.

#### Extremely randomized trees (Extra-Trees)

Extra-Trees averages the predictions from multiple decision trees, obtained by portioning the input-space with randomly generated splits^111^. However, Extra-Trees work on the full training set and select both the splitting feature and the split point at random^60^. Empirical work indicates that in high-dimensional or noisy settings Extra-Trees may match or exceed the performance of Random Forests.

#### Neural network (NeuralNetTorch)

Neural network consists of multiple layers of perceptrons or neurons, which learn to transform input data into desired output through a process of weighted connections. It utilizes backpropagation to adjust the weights based on the error between predicted and actual outputs, which facilitates learning intricate patterns in data. PyTorch implementation of Neural Network is used in this analysis^112^.

#### Quantum variational circuit (QVC)

**Fig. 1:**
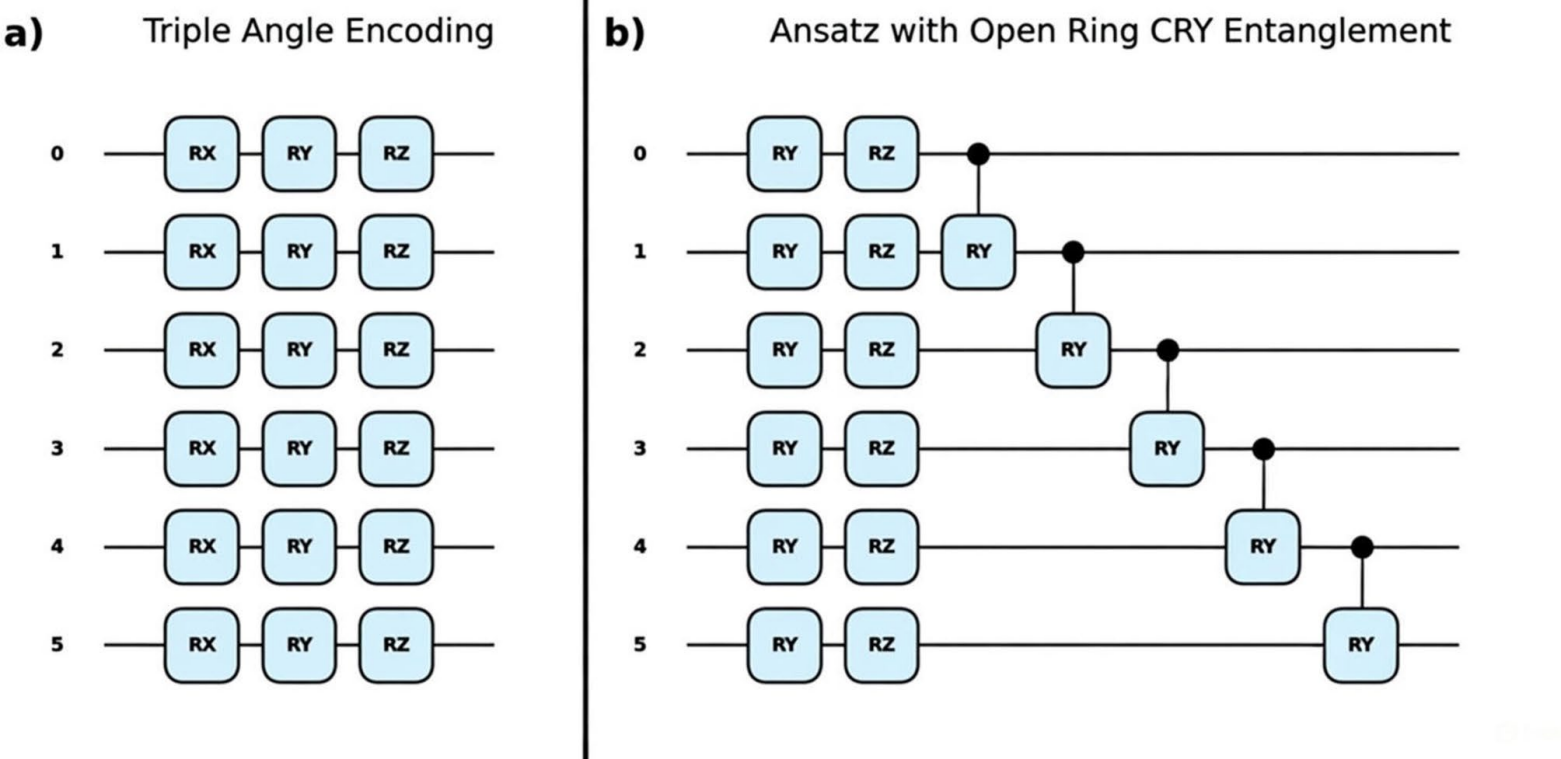
(**a**) Data encoder layer and (**b**) Ansatz of the quantum variational circuit.

QVCs encode classical data into quantum states and employ a parameterized quantum circuit (ansatz) to produce the predictions^93^. The data encoder circuit determines the frequency spectrum of the quantum model, which in turn affects its expressivity and thereby its ability to learn different types of functions^76^. In this study, a feature encoder with learnable parameters is used, as shown in Fig. 1, for the chosen input predictors ${x}_{m}$. The inputs are encoded through parameterized rotations ${R}_{X}\left({\theta }_{mX}\cdot {x}_{m}\right)$, ${R}_{Y}\left({\theta }_{mY}\cdot {x}_{m}\right)$ and ${R}_{Z}\left({\theta }_{mZ}\cdot {x}_{m}\right)$, as it has been shown that angle encoding with learnable parameters can help reduce circuit depth^113^. ${\theta }_{mX}$, ${\theta }_{mY}$ and ${\theta }_{mZ}$ are the learnable rotation parameters corresponding to the input feature ${x}_{m}$.

## Results and discussion

As already mentioned, it is not straightforward to evaluate the quality of ${GHI}_{CS}$ estimates in all-sky situations because ${GHI}_{CS}$ cannot be directly measured in cloudy situations. Therefore, the ${GHI}_{CS}$ estimates obtained from the ML models are evaluated by using them in the Heliosat-3 method and validating the accuracy of satellite-estimated all-sky GHI derived from them against the ground measured GHI. ${GHI}_{CS}$ from the physics-based McClear model, which utilizes CAMS AOD, is also used in the Heliosat-3 method to produce satellite-estimated all-sky GHI, and is used as a reference benchmark. All GHI datasets are averaged to 30 min resolution, prior to validation.

The general performance for all the test datapoints used in this analysis is evaluated using the coefficient of determination (R^2^), the root mean square error (RMSE), the mean absolute error (MAE) and the mean bias error (MBE), shown in Eqs. 5, 6, 7 and 8 respectively. The R^2^ metric gives an idea about the overall fit of the estimated values compared to the measured values. RMSE shows the average deviation of the estimated values with strong emphasis on large errors. The utility of the additional METAR data is analyzed by evaluating the percentage improvement in RMSE due to the ML models in comparison to the McClear model, across the available range of visibility values.

5 $${R}^{2}=1-{\sum }_{i=1}^{n}\frac{{\left({y}_{target}^{i}-{y}_{model}^{i}\right)}^{2}}{{\left({y}_{target}^{i}-\frac{1}{n}{\sum }_{i=1}^{n}{y}_{target}^{i}\right)}^{2}}$$

6 $$RMSE =\sqrt{\frac{1}{n}{\sum }_{i=1}^{N}{\left({y}_{model}^{i}-{y}_{target}^{i}\right)}^{2}}$$

7 $$MAE =\frac{1}{n}{\sum }_{i=1}^{N}\left|{y}_{model}^{i}-{y}_{target}^{i}\right|$$

8 $$MBE =\frac{1}{n}{\sum }_{i=1}^{N}\left({y}_{model}^{i}-{y}_{target}^{i}\right)$$

**Fig. 2:**
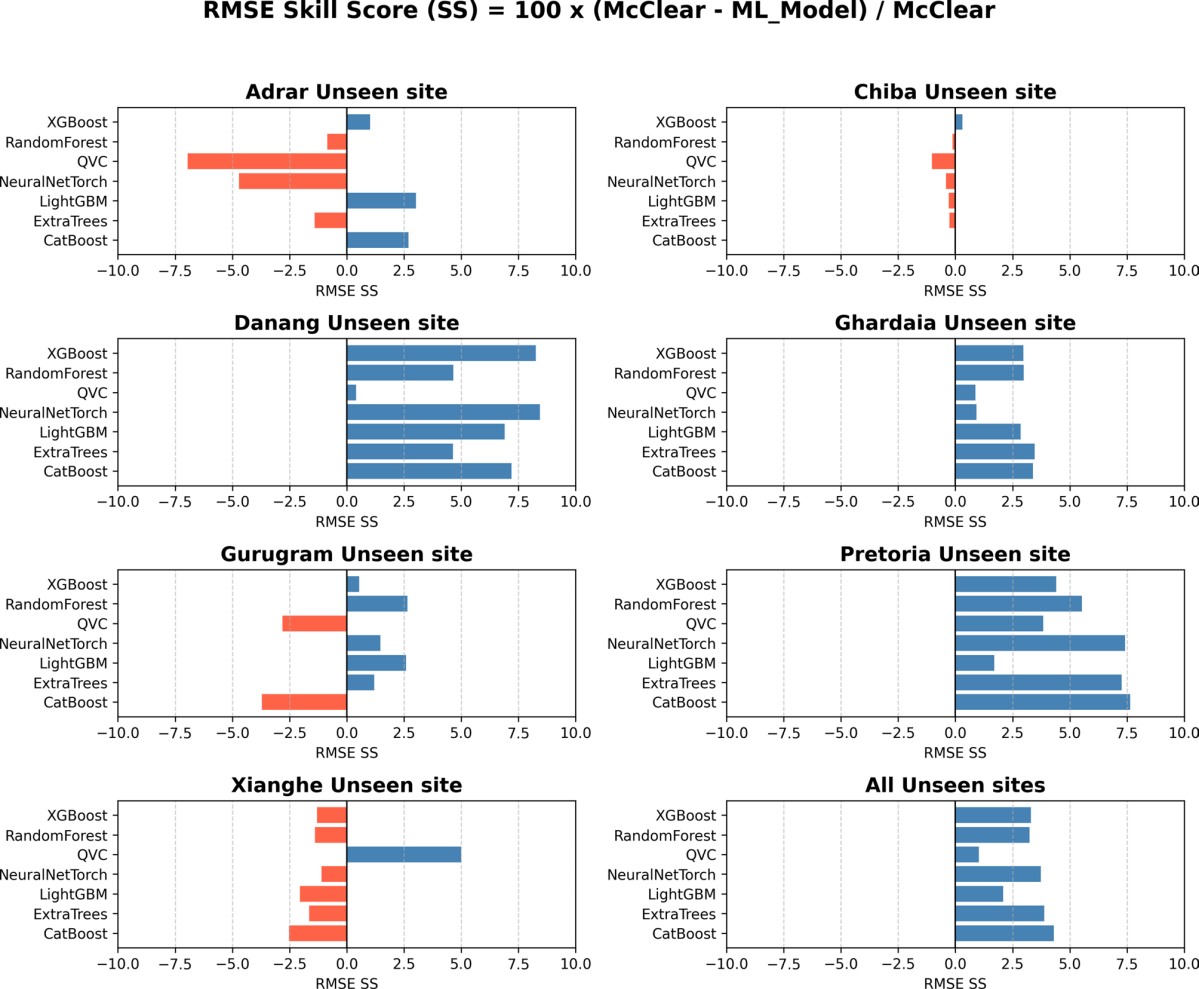
RMSE SS of Heliosat-3 estimated GHI when using ${GHI}_{CS}$ from the ML models instead of McClear. Blue implies positive SS or improvement and red implies negative SS or deterioration. Validation period: Chiba (2015–2017), Danang (2017–2019), Gurugram (2018–2019), Xianghe (2010–2015), Adrar (2015–2016), Ghardaia (2015–2018), Pretoria (2015–2024).

The overall all-sky RMSE in Heliosat-3 estimated GHI using the ${GHI}_{CS}$ values obtained from ML models, are slightly reduced compared to the RMSE when ${GHI}_{CS}$ obtained from McClear is used (Fig. 2). Out of the models tested in this study, CatBoost shows the highest RMSE Skill Score (SS) on an overall basis. While the QVC shows the least RMSE SS, it must also be considered that it uses a very low number of learnable parameters (188) in comparison to the other models such as the Neural Network (which uses 50561 learnable parameters). Also, the number of layers had to be restricted due to the computational requirements. Most of the ML models did not perform well at the Xinaghe and Chiba sites. The visibility measurement station is inside the Beijing airport close to the city while the GHI measurement station is 71 km away in Xianghe (shown in Table 1), which is less urbanized. The large distance and the difference in built environment presumably results in lower correlation in atmospheric aerosol composition and concentration between the two sites^114^. Similarly for the Chiba site, only the XGBoost model showed improvement relative to McClear. Here again, the visibility measurement site is inside Tokyo airport, which is located in the highly urbanized Inner Bay area. The GHI measurement site is located in Chiba, which has significantly lower built-up area compared to Tokyo Inner Bay^115^. Considerable differences in aerosol type and composition have been reported between these two areas^116^. The relatively poor performance of the ML models at the Xianghe and Chiba sites could therefore be attributed to the differences in micro-climate between the visibility and GHI measurement sites for these two locations, which leads to lower correlation in atmospheric aerosol composition and concentration. The R^2^ metric (Table 6) shows that the accuracy of the all-sky GHI derived using different ML models and McClear, are comparable. The overall impact is low, but positive.

**Table 6:** R^2^ of the satellite-estimated GHI against ground measured GHI using ${\mathrm{GHI}}_{\mathrm{CS}}$ from different models.

CatBoost	ExtraTrees	LightGBM	NeuralNet	QVC	RandomForest	XGBoost	McClear
0.93	0.93	0.92	0.93	0.92	0.93	0.93	0.92

From Table 7, it can be observed that the MBE in Heliosat-3 estimated GHI using the ${\mathrm{GHI}}_{\mathrm{CS}}$ values obtained from all the ML models except QVC, are slightly reduced compared to the MBE when ${\mathrm{GHI}}_{\mathrm{CS}}$ obtained from McClear is used. Most of the ML models that showed a positive RMSE SS in (Fig. 2), also showed a lower or comparable MBE than the reference McClear in (Table 7). The only exception is at the Adrar site. QVC showed the largest MBE on an overall basis.

**Table 7:** MBE of the satellite-estimated GHI against ground measured GHI using ${\mathrm{GHI}}_{\mathrm{CS}}$ from different models (in W/m2).

ML model	Chiba	Danang	Gurgaon	Xianghe	Adrar	Ghardaia	Pretoria	All unseen sites
XGBoost	-5.5	6.7	4.2	4.0	-12.3	-3.4	-12.7	-6.2
RandomForest	1.2	23.4	22.0	9.1	-12.1	-3.7	-8.8	-1.1
QVC	6.9	-32.1	-21.0	-4.2	2.9	-9.5	-29.9	-16
NeuralNetTorch	-10.4	5.0	15.4	7.6	-17.3	-12	-7.4	-5.4
LighGBM	-5.4	6.3	6.8	8.6	-9.2	-3.1	-15.1	-6.2
ExtraTrees	-1.3	23.6	28.2	12.3	-16.1	-5.7	-5.7	0
CatBoost	-4.4	7.7	1.9	1.1	-11.4	-3.5	-0.9	-1.5
Reference McClear	7.8	33.5	8.7	-17.1	-2.9	9.5	29.9	15.5

**Fig. 3:**
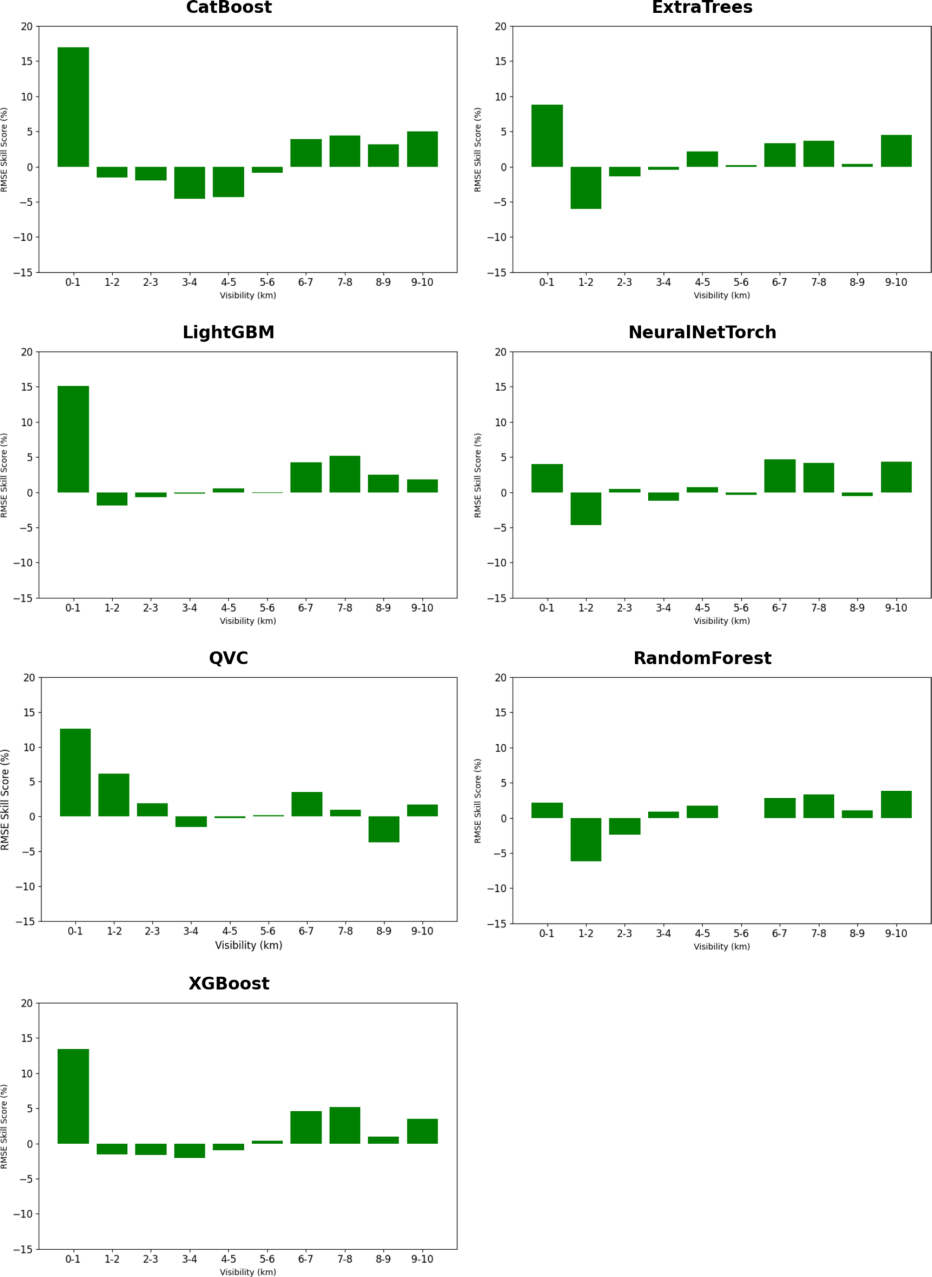
RMSE SS of Heliosat-3 estimated GHI when using ${GHI}_{CS}$ from the ML models instead of McClear for different visibility ranges at the seven unseen sites. Positive values of SS indicate improvement, while negative values indicate deterioration, with respect to McClear. Validation period: Chiba (2015–2017), Danang (2017–2019), Gurugram (2018–2019), Xianghe (2010–2015), Adrar (2015–2016), Ghardaia (2015–2018), Pretoria (2015–2024).

**Fig. 4:**
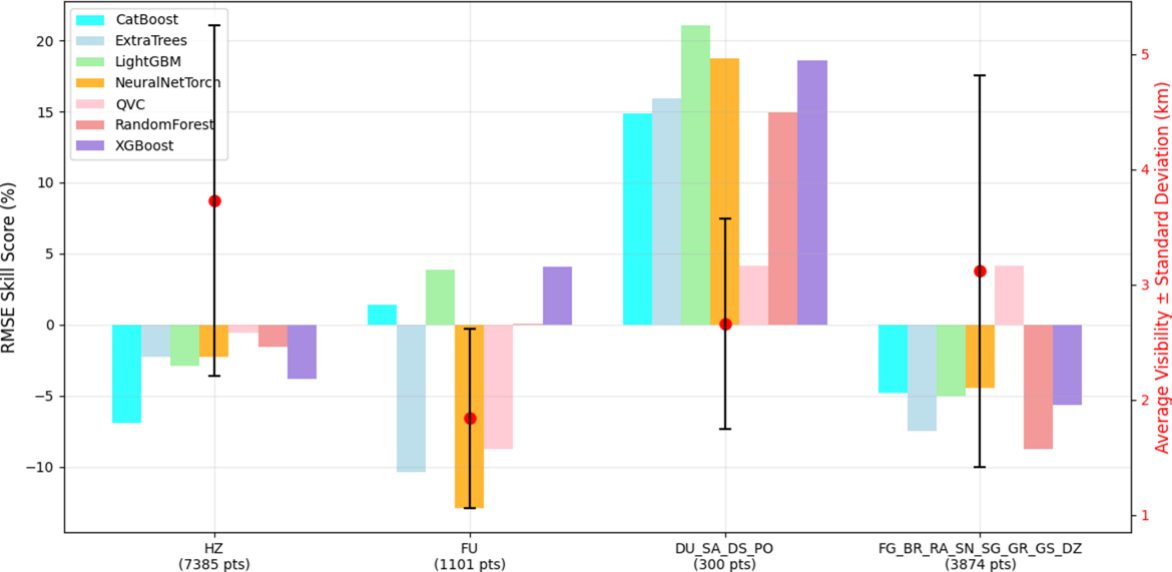
RMSE SS of Heliosat-3 estimated GHI when using ${GHI}_{CS}$ from the ML models instead of McClear in four different weather categories. HZ = Haze; FU = Smoke; DU,SA,DS,SS,PO = Widespread Dust, Sand, Duststorm, Sandstorm, Dust/Sand whirls; FG,BR,RA,SN,SG,GR,GS,DZ = Fog, Mist, Rain, Snow, Snow grains, Hail, Small hail, Drizzle . Positive values of SS indicate improvement, while negative values indicate deterioration, with respect to McClear. Validation period: Chiba (2015–2017), Danang (2017–2019), Gurugram (2018–2019), Xianghe (2010–2015), Adrar (2015–2016), Ghardaia (2015–2018), Pretoria (2015–2024).

Consistent positive RMSE SS is observed for the visibility ranges 0 to 1 km, 6 to 7 km and 9 to 10 km across all the models (Fig. 3). For the 7 to 8 km range, a positive RMSE SS is show by all the models except QVC and NeuralNetTorch. 10 km is the operational threshold of visibility reporting at airports, beyond which no significant weather phenomena such as haze, smog, dust storm, smoke etc., are found according to WMO guidelines^117^. However, it is also noticeable that for visibility values between 1 and 6 km, limited improvement or deterioration is observed in most cases. This could be attributed to the fact that most of the ML models showed a negative RMSE SS in weather situations with haze, precipitation and smoke, which correspond to the datapoints within the 1 – 6 km visibility range (see Fig. 4).

Figure 4 shows the RMSE SS in Heliosat-3 estimated GHI, when using ${GHI}_{CS}$ values from the ML models instead of the physics-based McClear model, for aerosol-relevant significant weather situations classified in the METAR data. The largest and most consistent positive RMSE SS is observed in the presence of dust and sand aerosol with all ML models. In particular, the LightGBM model shows the highest reduction in RMSE (approximately 21%). Only three models – CatBoost, LightGBM and XGBoost, show a significant reduction of RMSE during smoke events. While none of the ML models was able to show an improvement in RMSE during situations with haze. The lowest visibility values, ranging from 1 to 3 km, are observed for weather situations with smoke (FU). Smoke particles are typically small. This leads to a more effective extinction of light in the shorter wavelengths, leading to a greater reduction of visibility^118^. Dust particles, which are often larger^119^, tend to scatter light less efficiently but can still cause significant attenuation in high concentrations. Depending on the traveling distance, larger particles are removed by dry deposition. This explains the larger range of visibility values, between 2.5 and 5.5 km, observed in the presence of dust and sand aerosol events. Haze (HZ) primarily consists of dispersed secondary aerosols, which could also originate from anthropogenic sources as well as from biomass burning^120^. Due to the relatively lower concentrations than smoke at the source of origin, higher average visibility is observed during haze conditions in (Fig. 4). For the fourth category of hydrometeor related weather events (FG_BR_RA_SN_SG_GR_GS_DZ), all the models show a negative RMSE SS except QVC. This could be due to the fact that the cloud sources of hydrometeors are already being taken into account by the CI parameter, the lower visibility values may overcompensate for the reduction in GHI. Although, the RH parameter is used as an input in order to eliminate such situations, the filtering may not have been effective enough. Large errors in visibility derived AOD in situations with higher RH were noted in^37^. In general, the observations in this study are in line with previous findings that show that visibility is not a perfect proxy for AOD^121^.

**Fig. 5:**
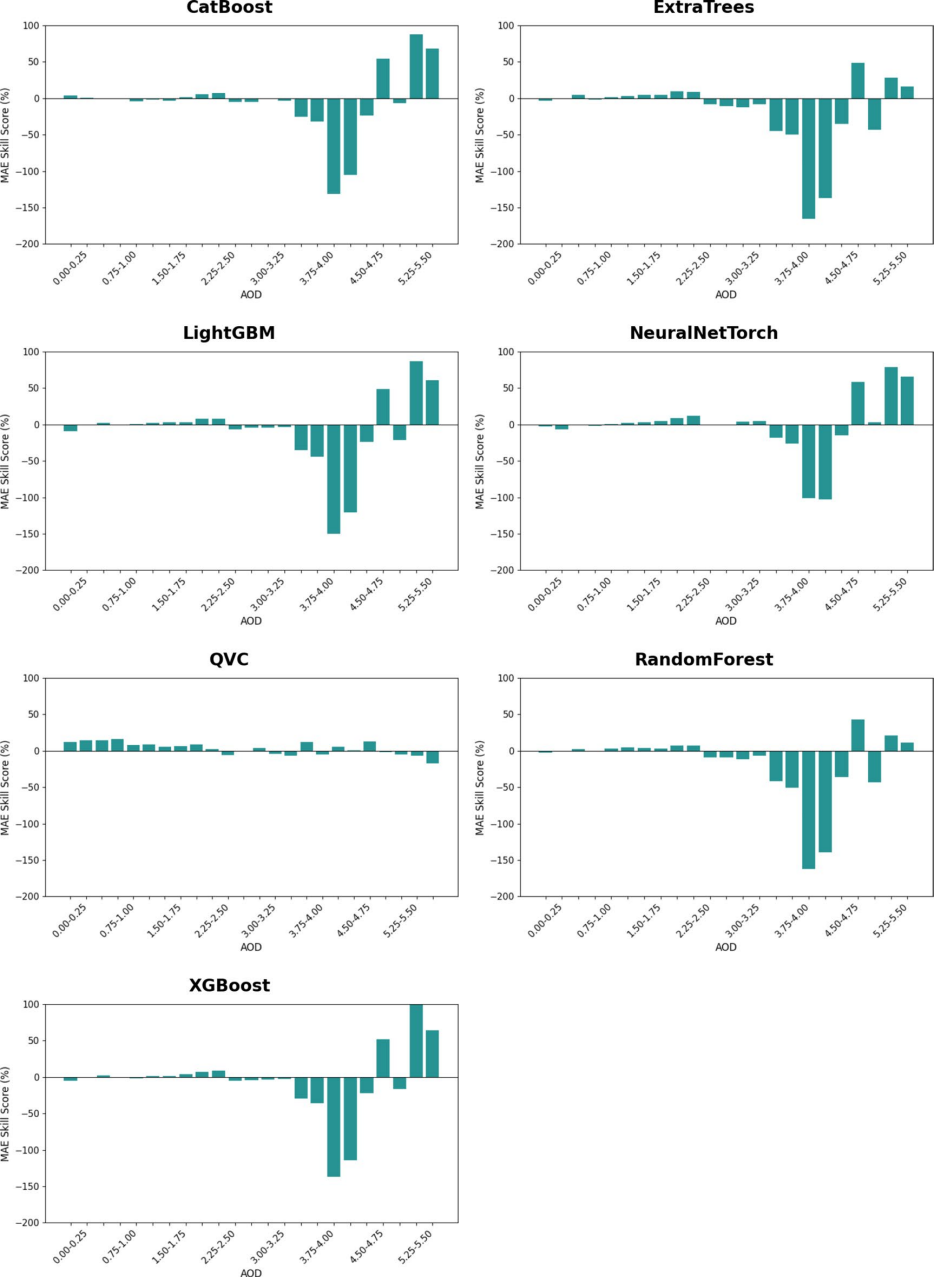
MAE SS of Heliosat-3 estimated GHI when using ${GHI}_{CS}$ from the ML models instead of McClear for different AOD ranges at the seven unseen sites. Positive values of SS indicate improvement, while negative values indicate deterioration, with respect to McClear. Validation period: Chiba (2015–2017), Danang (2017–2019), Gurugram (2018–2019), Xianghe (2010–2015), Adrar (2015–2016), Ghardaia (2015–2018), Pretoria (2015–2024).

The largest positive MAE Skill Scores (SS) of the Heliosat-3 estimated GHI, when using ${GHI}_{CS}$ values from the ML models instead of the physics-based McClear model, are observed for extremely high CAMS AOD values exceeding 4.75 for most of the models (Fig. 5). Such high values of AOD are known to occur during dust storms^122^. However, it should also be noted that the largest negative MAE SS are also observed for high values of AOD (> 3.25). The only exception is QVC, which exhibits relatively small positive or negative MAE SS compared to the other ML models. It is also interesting to note that positive MAE SS values are also observed for some intermediate AOD ranges in all the models.

## Summary and conclusion

This study introduced a machine learning (ML) framework for estimating global horizontal clear sky irradiance (${GHI}_{CS}$) at 30-min resolution by combining atmospheric parameters from the METeorological Aerodrome Report (METAR) with aerosol information from Copernicus Atmosphere Monitoring Service (CAMS) reanalysis. To address the absence of direct ${GHI}_{CS}$ measurements, a normalized pseudo global horizontal clear sky irradiance (${GHI}_{CS}^{*}$) target was employed for model training. Models trained on data from Cairo were tested on seven unseen sites in tropical and sub-tropical environments.

When coupled with the Heliosat-3 model to derive all-sky GHI, the ML-derived ${GHI}_{CS}$ values outperformed the physics-based McClear estimates on an overall basis. Categorical boosting (CatBoost) yielded the most robust overall improvement in terms of RMSE SS, while quantum variational circuit (QVC) achieved notable gains despite the limited number of parameters. The most consistent improvements were observed for visibility values between 6 and 8 km. Large reductions in RMSE of up to 21% were observed during dust and sand aerosol events, with moderate improvements under smoke, while haze events showed no improvement. The largest improvements as well as deteriorations were observed for CAMS AOD values exceeding 3, except in the case of the Quantum Variational Circuit (QVC). Additionally, all models showed improvement for some intermediate CAMS AOD ranges as well.

These findings demonstrate that ML-based ${GHI}_{CS}$ estimates using local METAR data offer a useful enhancement for the existing satellite-based GHI estimation models, particularly in aerosol-rich regions where existing physics-based models face limitations due to spatial resolution. Looking ahead, expanding the training domain to include fractions of data from multiple sites, incorporating aerosol-type specific AOD, especially for dust, including information on boundary layer height and exploring domain adaptation techniques may further improve the accuracy of satellite retrieved GHI. This approach holds promise for advancing operational PV power prediction and solar resource assessment in regions strongly impacted by aerosols.

## Data Availability

SARAH3, METAR, CAMS global reanalysis and ground observations used in this study are openly available for any purpose, SOLCAST data is available for research and education purposes. All download links are mentioned in the data section. The output data of the machine learning models will be made available upon request.

## References

[1] Al-Dahidi, S., Ayadi, O., Alrbai, M. & Adeeb, J. Ensemble approach of optimized artificial neural networks for solar photovoltaic power prediction. *IEEE Access* **7**, 81741–81758 (2019).

[2] Khodayar, M., Mohammadi, S., Khodayar, M. E., Wang, J. & Liu, G. Convolutional graph Autoencoder: A generative deep neural network for probabilistic spatio-temporal solar irradiance forecasting. *IEEE Trans. Sustain. Energy* **11**, 571–583 (2020).

[3] Najdawi, F. Z. & Villarreal, R. Utilizing the Vector autoregression model (VAR) for short-term solar irradiance forecasting. *EPE* **15**, 353–362 (2023).

[4] Edoli, E., Fiorenzani, S. & Vargiolu, T. *Optimization Methods for Gas and Power Markets* (Palgrave Macmillan, 2016).

[5] Dunlop, E. D., Wald, L. & Suri, M. *Solar Energy Resource Management for Electricity Generation from Local Level to Global Scale* (Nova Science Publishers Inc, 2006).

[6] Yamasoe, M. A., do Rosário, N. M. E. & Barros, K. M. Downward solar global irradiance at the surface in São Paulo city—The climatological effects of aerosol and clouds. *JGR Atmos.* **122**, 391–404 (2017).

[7] Kosmopoulos, P. G. et al. Dust impact on surface solar irradiance assessed with model simulations, satellite observations and ground-based measurements. *Atmos. Meas. Tech.* **10**, 2435–2453 (2017).

[8] Schafer, J. S. et al. Observed reductions of total solar irradiance by biomass-burning aerosols in the Brazilian Amazon and Zambian Savanna. *Geophys. Res. Lett.* **29**, 1–4 (2002).

[9] Costa, R. S., Martins, F. R. & Pereira, E. B. Atmospheric aerosol influence on the Brazilian solar energy assessment: Experiments with different horizontal visibility bases in radiative transfer model. *Renew. Energy* **90**, 120–135 (2016).

[10] Husar, R. B., Husar, J. D. & Martin, L. Distribution of continental surface aerosol extinction based on visual range data. *Atmos. Environ.* **34**, 5067–5078 (2000).

[11] Tantiwechwuttikul, R., Yarime, M. & Ito, K. *Technologies and Eco-innovation towards Sustainability II* (Springer, 2019).

[12] Hermann, M. et al*.* Meridional distributions of aerosol particle number concentrations in the upper troposphere and lower stratosphere obtained by civil aircraft for regular investigation of the atmosphere based on an instrument container (CARIBIC) flights. *J. Geophys. Res.* **108** (2003).

[13] Sun, X. et al. Worldwide performance assessment of 95 direct and diffuse clear-sky irradiance models using principal component analysis. *Renew. Sustain. Energy Rev.* **135**, 110087 (2021).

[14] Kamath, H. G. & Srinivasan, J. Validation of global irradiance derived from INSAT-3D over India. *Sol. Energy* **202**, 45–54 (2020).

[15] Gueymard, C. A., Habte, A. & Sengupta, M. Reducing uncertainties in large-scale solar resource data: The impact of aerosols. *IEEE J. Photovolt.* **8**, 1732–1737 (2018).

[16] Foyo-Moreno, I. et al. Estimating aerosol characteristics from solar irradiance measurements at an urban location in southeastern Spain. *JGR Atmos.* **119**, 1845–1859 (2014).

[17] Houborg, R., Soegaard, H., Emmerich, W. & Moran, S. Inferences of all-sky solar irradiance using Terra and Aqua MODIS satellite data. *Int. J. Remote Sens.* **28**, 4509–4535 (2007).

[18] Remund, J., Wald, L., Lefèvre, M., Ranchin, T. & Page, J. H. *Proceedings of ISES Solar World Congress 2003* pp. 13 (International Solar Energy Society (ISES), 2003).

[19] Kim, M., Levy, R. C., Remer, L. A., Mattoo, S. & Gupta, P. Parameterizing spectral surface reflectance relationships for the dark target aerosol algorithm applied to a geostationary imager. *Atmos. Meas. Tech.* **17**, 1913–1939 (2024).

[20] Schutgens, N. A. J. Site representativity of AERONET and GAW remotely sensed aerosol optical thickness and absorbing aerosol optical thickness observations. *Atmos. Chem. Phys.* **20**, 7473–7488 (2020).

[21] Lee, K.-H., Yoo, J.-M. & Wong, M.-S. in *2020 IEEE International Geoscience & Remote Sensing Symposium* pp. 5600–5603. (IEEE, 2020).

[22] Lefèvre, M. et al. McClear: a new model estimating downwelling solar radiation at ground level in clear-sky conditions. *Atmos. Meas. Tech.* **6**, 2403–2418 (2013).

[23] Gueymard, C. A. & Yang, D. Worldwide validation of CAMS and MERRA-2 reanalysis aerosol optical depth products using 15 years of AERONET observations. *Atmos. Environ.* **225**, 117216 (2020).

[24] Isaza, A., Kay, M., Evans, J. P., Bremner, S. & Prasad, A. Validation of Australian atmospheric aerosols from reanalysis data and CMIP6 simulations. *Atmos. Res.* **264**, 105856 (2021).

[25] Ansari, K. & Ramachandran, S. Optical and physical characteristics of aerosols over Asia: AERONET, MERRA-2 and CAMS. *Atmos. Environ.* **326**, 120470 (2024).

[26] Witthuhn, J., Hünerbein, A. & Deneke, H. Evaluation of satellite-based aerosol datasets and the CAMS reanalysis over ocean utilizing shipborne reference observations. *Atmos. Meas. Tech.* **13**, 1387–1412 (2020).

[27] Júnior, A. L. P. et al. Evaluation of aerosol optical depth (Aod) estimated by copernicus atmosphere monitoring service (Cams) in Brazil. *Theor. Appl. Climatol.* **156**, 116 (2025).

[28] Tuna Tuygun, G. & Elbir, T. Comparative analysis of CAMS aerosol optical depth data and AERONET observations in the Eastern Mediterranean over 19 years. *Environ. Sci. Pollut. Res.* **31**, 27069–27084 (2024). 10.1007/s11356-024-32950-6 PMC11052789 38503950

[29] Koschmieder, H. Theorie der horizontalen sichtweite, Beitrage zur Physik der Freien Atmosphare. *Meteorol. Z.* **12**, 3353 (1924).

[30] Horvath, H. On the applicability of the koschmieder visibility formula. *Atmos. Environ.* **1967**(5), 177–184 (1971).

[31] Ozkaynak, H., Schatz, A. D., Thurston, G. D., Isaacs, R. G. & Husar, R. B. Relationships between aerosol extinction coefficients derived from airport visual range observations and alternative measures of airborne particle mass. *J. Air Pollut. Control Assoc.* **35**, 1176–1185 (1985).

[32] Friedlander, S. K. *Smoke, Dust and Haze* (Oxford University Press, 2000).

[33] Peterson, J. T. & Fee, C. J. Visibility-atmospheric turbidity dependence at Raleigh, North Carolina. *Atmos. Environ.* **1967**(15), 2561–2563 (1981).

[34] Elterman, L. Relationships between vertical attenuation and surface meteorological range. *Appl. Opt.* **9**, 1804–1810 (1970). 20094141 10.1364/AO.9.001804

[35] Zhang, S., Wu, J., Fan, W., Yang, Q. & Zhao, D. Review of aerosol optical depth retrieval using visibility data. *Earth-Sci. Rev.* **200**, 102986 (2020).

[36] Qiu, J. & Lin, Y. A parameterization model of aerosol optical depths in China. *Acta Meteorol. Sin.* **59**, 368–372 (2001).

[37] Wu, J. et al. Improvement of aerosol optical depth retrieval using visibility data in China during the past 50 years. *JGR Atmos.* **119**, 13–370 (2014).

[38] Zhang, Z. et al. Aerosol optical depth retrieval from visibility in China during 1973–2014. *Atmos. Environ.* **171**, 38–48 (2017).

[39] Li, F. et al. An improved method for retrieving aerosol optical depth using the ground-level meteorological data over the South-central Plain of Hebei Province, China. *Atmos. Pollut. Res.* **13**, 101334 (2022).

[40] Wu, J. et al. Using particle swarm optimization to improve visibility-aerosol optical depth retrieval method. *NPJ Clim. Atmos. Sci.* **4**, 1–12 (2021).

[41] World Meteorological Organization. Manual on Codes: International Codes Volume I.1, Annex II to the WMO Technical Regulations, Part A ‐ Alphanumeric Codes. Technical Manual. World Meteorological Organization, 2019

[42] International Civil Aviation Organization. Annex 3 to the Convention on International Civil Aviation: Meteorological Service for International Air Navigation. Standard. International Civil Aviation Organization, (2018).

[43] Hao, H., Wang, K., Zhao, C., Wu, G. & Li, J. Visibility-derived aerosol optical depth over global land from 1959 to 2021. *Earth Syst. Sci. Data* **16**, 3233–3260 (2024).

[44] Vijayakumar, K. et al. Solar radiometer sensing of multi-year aerosol features over a tropical urban station: direct-Sun and inversion products. *Atmos. Meas. Tech.* **13**, 5569–5593 (2020).

[45] Ineichen, P. & Perez, R. Aerosol quantification based on global irradiance. *Solar Paces 2010 proceedings* (2010).

[46] Sequeira, R. & Lai, K.-H. The effect of meteorological parameters and aerosol constituents on visibility in urban Hong Kong. *Atmos. Environ.* **32**, 2865–2871 (1998).

[47] Wen, C.-C. & Yeh, H.-H. Comparative influences of airborne pollutants and meteorological parameters on atmospheric visibility and turbidity. *Atmos. Res.* **96**, 496–509 (2010).

[48] Peng, Y. et al. Improved method of visibility parameterization focusing on high humidity and aerosol concentrations during fog–haze events: Application in the GRAPES_CAUCE model in Jing-Jin-Ji, China. *Atmos. Environ.* **222**, 117139 (2020).

[49] Goudie, A. S. & Middleton, N. J. The changing frequency of dust storms through time. *Clim. Change* **20**, 197–225 (1992).

[50] Mahowald, N. M., Ballantine, J. A., Feddema, J. & Ramankutty, N. Global trends in visibility: implications for dust sources. *Atmos. Chem. Phys.* **7**, 3309–3339 (2007).

[51] Tavartkiladze, K. A. & Amiranashvili, A. G. *Nucleation and Atmospheric Aerosols* (Springer, 2007).

[52] Wilson, R. T., Milton, E. J. & Nield, J. M. Are visibility-derived AOT estimates suitable for parameterizing satellite data atmospheric correction algorithms?. *Int. J. Remote Sens.* **36**, 1675–1688 (2015).

[53] Verbois, H., Rusydi, A. & Thiery, A. Probabilistic forecasting of day-ahead solar irradiance using quantile gradient boosting. *Sol. Energy* **173**, 313–327 (2018).

[54] Nabavi, S. O., Haimberger, L., Abbasi, R. & Samimi, C. Prediction of aerosol optical depth in West Asia using deterministic models and machine learning algorithms. *Aeolian Res.* **35**, 69–84 (2018).

[55] Kosmopoulos, P. *Planning and Management of Solar Power from Space* (Academic Press, 2024).

[56] Neher, I., Buchmann, T., Crewell, S., Pospichal, B. & Meilinger, S. Impact of atmospheric aerosols on solar power. *Meteorol. Z.* **28**, 305–321 (2019).

[57] Bentéjac, C., Csörgő, A. & Martínez-Muñoz, G. A comparative analysis of gradient boosting algorithms. *Artif. Intell. Rev.* **54**, 1937–1967 (2021).

[58] Zhang, J., Mucs, D., Norinder, U. & Svensson, F. LightGBM: An effective and scalable algorithm for prediction of chemical toxicity-application to the Tox21 and mutagenicity data sets. *J. Chem. Inf. Model.* **59**, 4150–4158 (2019). 31560206 10.1021/acs.jcim.9b00633

[59] Hancock, J. T. & Khoshgoftaar, T. M. CatBoost for big data: an interdisciplinary review. *J. Big Data* **7**, 94 (2020). 33169094 10.1186/s40537-020-00369-8 PMC7610170

[60] Geurts, P., Ernst, D. & Wehenkel, L. Extremely randomized trees. *Mach. Learn.* **63**, 3–42 (2006).

[61] Car, Z., Baressi Šegota, S., Anđelić, N., Lorencin, I. & Mrzljak, V. Modeling the spread of COVID-19 infection using a multilayer perceptron. *Comput. Math. Methods Med.* **2020**, 5714714 (2020). 32565882 10.1155/2020/5714714 PMC7260624

[62] Benedetti, M., Lloyd, E., Sack, S. & Fiorentini, M. Parameterized quantum circuits as machine learning models. *Quantum Sci. Technol.* **4**, 43001 (2019).

[63] Schuld, M., Sinayskiy, I. & Petruccione, F. An introduction to quantum machine learning. *Contemp. Phys.* **56**, 172–185 (2015).

[64] Blanc, P., Jolivet, R., Ménard, L. & Saint-Drenan, Y.-M. Data sharing of in-situ measurements following GEO and FAIR principles in the solar energy sector. *Centre O.I.E. MINES Paris, Working document* (2022).

[65] Driemel, A. et al. Baseline surface radiation network (BSRN): structure and data description (1992–2017). *Earth Syst. Sci. Data* **10**, 1491–1501 (2018).

[66] Jolivet, R. & Saint-Drenan, Y. M. *libinsitu: A library to transform solar in situ data into a standard NetCDF format* https://git.sophia.minesparis.psl.eu/oie/libinsitu. (2022).

[67] El‐Metwally, M., Alfaro, S. C., Abdel Wahab, M. & Chatenet, B. Aerosol characteristics over urban Cairo: Seasonal variations as retrieved from Sun photometer measurements. *JGR Atmospheres* **113** (2008).

[68] El-Askary, H. & Kafatos, M. Dust storm and black cloud influence on aerosol optical properties over Cairo and the Greater Delta region, Egypt. *Int. J. Remote Sens.* **29**, 7199–7211 (2008).

[69] Christodoulou, A. et al. *Submicron Aerosol Pollution in Greater Cairo (Egypt): A New Type of Urban Haze?* (Copernicus GmbH, 2024). 10.1016/j.envint.2024.108610 38626495

[70] Lalchandani, V. et al. Effect of biomass burning on PM 2.5 composition and secondary aerosol formation during post-monsoon and winter haze episodes in Delhi. *JGR Atmosph.* **127**, e2021JD035232 (2022).

[71] Bhowmik, H. S. et al. Contribution of fossil and biomass-derived secondary organic carbon to winter water-soluble organic aerosols in Delhi. *India. Sci. Total Environ.* **912**, 168655 (2024). 37992837 10.1016/j.scitotenv.2023.168655

[72] Jain, S., Sharma, S. K., Vijayan, N. & Mandal, T. K. Seasonal characteristics of aerosols (PM2.5 and PM10) and their source apportionment using PMF: A four year study over Delhi India. *Environ. Pollut. (Barking, Essex: 1987)* **262**, 114337 (2020). 10.1016/j.envpol.2020.114337 32193082

[73] Sharma, M., Kaskaoutis, D. G., Singh, R. P. & Singh, S. Seasonal variability of atmospheric aerosol parameters over greater noida using ground sunphotometer observations. *Aerosol. Air Qual. Res.* **14**, 608–622 (2014).

[74] Yan, L. & Liu, X. Seasonal variation of atmospheric aerosol and its relation to cloud faction over Beijing-Tianjin-Hebei region. *Chin. Res. Environ. Sci* **22**, 924–931 (2009).

[75] Li, B. G., Ran, Y. & Tao, S. Seasonal variation and spatial distribution of atmospheric aerosols in Beijing. *Acta Sci. Circumstant.* **28**, 1425–1429 (2008).

[76] Takegawa, N. et al. Seasonal and diurnal variations of submicron organic aerosol in Tokyo observed using the Aerodyne aerosol mass spectrometer. *JGR Atmospheres* **111** (2006).

[77] Iijima, A. et al. Regional and seasonal characteristics of emission sources of fine airborne particulate matter collected in the center and suburbs of Tokyo, Japan as determined by multielement analysis and source receptor models. *J. Environ. Monit.* **10**, 1025–1032 (2008). 18728894 10.1039/b806483k

[78] Phan, N.-T. & Dinh-Tri, C. Assessment of air pollutant emissions from rice straw open burning in Hoa Vang district, Da Nang city, Vietnam. *UD-JST,* 25–32 (2024).

[79] Pham, T. T. K., Le, S. H., Nguyen, T., Balasubramanian, R. & Tran, P. T. M. Characteristics of airborne particles in stone quarrying areas: Human exposure assessment and mitigation. *Environ. Res.* **245**, 118087 (2024). 38159664 10.1016/j.envres.2023.118087

[80] Jenkins, G. S. & Gueye, M. WRF 1960–2014 winter season simulations of particulate matter in the sahel: implications for air quality and respiratory health. *GeoHealth* **2**, 248–260 (2018). 32159017 10.1002/2018GH000132 PMC7007090

[81] Marif, Y. et al. Estimation of atmospheric turbidity over Adrar city in Algeria. *J. King Saud Univ. Sci.* **31**, 143–149 (2019).

[82] Djafer, D. & Irbah, A. Estimation of atmospheric turbidity over Ghardaïa city. *Atmos. Res.* **128**, 76–84 (2013).

[83] Mohamed, Z., Djelloul, D. & Fatima, C. Classification of aerosol types over Ghardaia, Algeria, based on MODIS data. *IJESD* **7**, 745 (2016).

[84] Howlett-Downing, C., Boman, J., Molnár, P., Shirinde, J. & Wichmann, J. PM2.5 chemical composition and geographical origin of air masses in Pretoria, South Africa. *Water Air Soil Pollut.* **233**, 271 (2022).

[85] Adesina, A. J., Kumar, K. R., Sivakumar, V. & Griffith, D. Direct radiative forcing of urban aerosols over Pretoria (25.75°S, 28.28°E) using AERONET Sunphotometer data: first scientific results and environmental impact. *J. Environ. Sci.* **26**, 2459–2474 (2014). 10.1016/j.jes.2014.04.006 25499494

[86] Pfeifroth, U. et al.* Surface Radiation Data Set - Heliosat (SARAH) - Edition 3* (Satellite Application Facility on Climate Monitoring (CM SAF), 2023), https://wui.cmsaf.eu/safira/action/viewDetails?acronym=SARAH_V003.

[87] Hammer, A. et al. Solar energy assessment using remote sensing technologies. *Remote Sens. Environ.* **86**, 423–432 (2003).

[88] Cano, D. et al. A method for the determination of the global solar radiation from meteorological satellite data. *Sol. Energy* **37**, 31–39 (1986).

[89] Rigollier, C., Lefèvre, M. & Wald, L. The method Heliosat-2 for deriving shortwave solar radiation from satellite images. *Sol. Energy* **77**, 159–169 (2004).

[90] Betcke, J. et al. Energy-specific solar radiation data from meteosat second generation (MSG): The Heliosat-3 Project. Final Report, (2005).

[91] Hammer, A., Kühnert, J., Weinreich, K. & Lorenz, E. Short-term forecasting of surface solar irradiance based on meteosat-SEVIRI data using a nighttime cloud index. *Remote Sens.* **7**, 9070–9090 (2015).

[92] Solar API and Weather Forecasting Tool | Solcast™. https://solcast.com/ (2025).

[93] Bright, J. M. Solcast: Validation of a satellite-derived solar irradiance dataset. *Sol. Energy* **189**, 435–449 (2019).

[94] Bright, J. M., Killinger, S., Lingfors, D. & Engerer, N. A. Integration of distributed solar forecasting with distribution network operations in Australia. *ISES Sol. World Congr. 2015, Abu Dhabi, United Arab Emirates, Oct. 29-Novemb. 2* (2017).

[95] ECMWF. CAMS solar radiation time-series: data documentation. Copernicus Knowledge Base - ECMWF Confluence Wiki. https://confluence.ecmwf.int/display/CKB/CAMS+solar+radiation+time-series%3A+data+documentation.

[96] Inness, A. et al. The CAMS reanalysis of atmospheric composition. *Atmos. Chem. Phys.* **19**, 3515–3556 (2019).

[97] ICAO. Annex 3 to the Convention on International Civil Aviation—Meteorological Service for International Air Navigation (2018).

[98] Herzmann, D., Arritt, R. & Todey, D. Iowa environmental mesonet. *mesonet. agron. iastate. edu/request/coop/fe. phtml (verified 27 Sept. 2005). Iowa State Univ., Dep. of Agron., Ames, IA* (2004).

[99] Chan, P. W. A test of visibility sensors at Hong Kong international airport. *Weather* **71**, 241–246 (2016).

[100] Bilbao, J., Román, R., Yousif, C., Mateos, D. & de Miguel, A. Total ozone column, water vapour and aerosol effects on erythemal and global solar irradiance in Marsaxlokk. *Malta. Atmos. Environ.* **99**, 508–518 (2014).

[101] Bright, J. M. et al. Bright-Sun: A globally applicable 1-min irradiance clear-sky detection model. *Renew. Sustain. Energy Rev.* **121**, 109706 (2020).

[102] Alia-Martinez, M., Antonanzas, J., Urraca, R., Martinez-de-Pison, F. J. & Antonanzas-Torres, F. Benchmark of algorithms for solar clear-sky detection. *J. Renew. Sustain. Energy* **8** (2016).

[103] Ellis, B. H., Deceglie, M. & Jain, A. Automatic detection of clear-sky periods from irradiance data. *IEEE J. Photovolt.* **9**, 998–1005 (2019).

[104] Roy, A. et al. Parallax and cloud shadow correction in satellite-based solar irradiance estimation: A study in tropical environments. *Appl. Energy* **399**, 126457 (2025).

[105] Zelenka, A., Perez, R., Seals, R. & Renné, D. Effective accuracy of satellite-derived hourly irradiances. *Theor. Appl. Climatol.* **62**, 199–207 (1999).

[106] Chen, T. & Guestrin, C. *Proceedings of the 22nd ACM SIGKDD International Conference on Knowledge Discovery and Data Mining* pp. 785–794. (Association for Computing Machinery, 2016).

[107] Shi, Y., Ke, G., Chen, Z., Zheng, S. & Liu, T.-Y. *Advances in Neural Information Processing Systems* (Curran Associates Inc, 2022).

[108] Prokhorenkova, L., Gusev, G., Vorobev, A., Dorogush, A. V. & Gulin, A. CatBoost: unbiased boosting with categorical features. *Adv. Neural Inf. Process. Syst.* **31** (2018).

[109] Sahin, E. K. Comparative analysis of gradient boosting algorithms for landslide susceptibility mapping. *Geocarto Int.* **37**, 2441–2465 (2022).

[110] Scornet, E. Random forests and kernel methods. *IEEE Trans. Inf. Theory* **62**, 1485–1500 (2016).

[111] Wehenkel, L., Ernst, D. & Geurts, P. *Robust methods for power system state estimation and load forecasting* (2006).

[112] Paszke, A. *et al.* PyTorch: An Imperative style, high-performance deep learning library. *Adv. Neural Inf. Process. Syst.* **32** (2019).

[113] Ovalle-Magallanes, E., Alvarado-Carrillo, D. E., Avina-Cervantes, J. G., Cruz-Aceves, I. & Ruiz-Pinales, J. Quantum angle encoding with learnable rotation applied to quantum–classical convolutional neural networks. *Appl. Soft Comput.* **141**, 110307 (2023).

[114] Wang, L. et al. Contrasting characteristics of the surface energy balance between the urban and rural areas of Beijing. *Adv. Atmos. Sci.* **32**, 505–514 (2015).

[115] Wang, R., Gao, W. & Peng, W. Spatial downscaling method for air temperature through the correlation between land use/land cover and microclimate: A case study of the Greater Tokyo Area, Japan. *Urban Clim.* **40**, 101003 (2021).

[116] Hanari, N. et al. Dioxin-like compounds in pine needles around Tokyo Bay, Japan in 1999. *J. Environ. Monit.* **6**, 305–312 (2004). 15054539 10.1039/b311176h

[117] *Manual on Codes, International Codes, vol. I. 1 (Annex II to WMO Technical Regulations), part A, Alphanumeric Codes* (1995).

[118] Yang, Y. & Li, T. *Fourth Seminar on Novel Optoelectronic Detection Technology and Application* pp. 165–171. (2018).

[119] Janicka, L., Stachlewska, I. S., Veselovskii, I. & Baars, H. Temporal variations in optical and microphysical properties of mineral dust and biomass burning aerosol derived from daytime Raman lidar observations over Warsaw, Poland. *Atmos. Environ.* **169**, 162–174 (2017).

[120] Guo, B. et al. Temporal and spatial variations of haze and fog and the characteristics of PM2.5 during heavy pollution episodes in China from 2013 to 2018. *Atmos. Pollut. Res.* **11**, 1847–1856 (2020).

[121] Zhang, Z. Y., Wong, M. S. & Lee, K. H. Evaluation of the representativeness of ground-based visibility for analysing the spatial and temporal variability of aerosol optical thickness in China. *Atmos. Environ.* **147**, 31–45 (2016).

[122] Alam, K., Trautmann, T., Blaschke, T. & Subhan, F. Changes in aerosol optical properties due to dust storms in the Middle East and Southwest Asia. *Remote Sens. Environ.* **143**, 216–227 (2014).

